# Design and Analysis of a Low-Voltage VCO: Reliability and Variability Performance

**DOI:** 10.3390/mi14112118

**Published:** 2023-11-18

**Authors:** Tayebeh Azadmousavi, Ebrahim Ghafar-Zadeh

**Affiliations:** 1 Department of Electrical Engineering, University of Bonab, Bonab 55517-61167, Iran; tayebehazadmousavi@gmail.com; 2 Biologically Inspired Sensors and Actuators (BioSA), Department of Electrical Engineering and Computer Science (EECS), Lassonde School of Engineering, York University, Toronto, ON M3J 1P3, Canada

**Keywords:** VCO, threshold voltage, electron mobility, low voltage, phase noise

## Abstract

This paper investigates an adaptive body biasing (ABB) circuit to improve the reliability and variability of a low-voltage inductor–capacitor (LC) voltage-controlled oscillator (VCO). The ABB circuit provides VCO resilience to process variability and reliability variation through the threshold voltage adjustment of VCO’s transistors. Analytical equations considering the body bias effect are derived for the most important relations of the VCO and then the performance is verified using the post-layout simulation results. Under a 0.16% threshold voltage shift, the sensitivity of the normalized phase noise and transconductance of the VCO with the ABB circuit compared to the constant body bias (CBB) decreases by around 8.4 times and 3.1 times, respectively. Also, the sensitivity of the normalized phase noise and transconductance of the proposed VCO under 0.16% mobility variations decreases by around 1.5 times and 1.7 times compared to the CBB, respectively. The robustness of the VCO is also examined using process variation analysis through Monte Carlo and corner case simulations. The post-layout results in the 180 nm CMOS process indicate that the proposed VCO draws a power consumption of only 398 µW from a 0.6 V supply when the VCO frequency is 2.4 GHz. It achieves a phase noise of −123.19 dBc/Hz at a 1 MHz offset and provides a figure of merit (FoM) of −194.82 dBc/Hz.

## 1. Introduction

Over the past decades, biosensors have become one of the most important analytical devices that serve as an indispensable platform for biotechnology and life science research. Much research focuses on expanding the evolving bio-sensing from off-site laboratory tests to research in the place of patient care or bedside laboratory tests that are referred to as Point-of-Care testing (PoC). The PoC platform enables easy-to-use, rapid diagnosis and a compact size at an affordable price, but these specifications make the construction of such a PoC device quite a challenge [1,2,3,4,5,6]. The major challenge that must be overcome to develop PoC devices is the monolithic integration of biochemical methods (assays) with biosensor arrays. Complementary metal–oxide–semiconductor (CMOS) technology that offers cost efficiency, low power consumption, and a significant degree of design flexibility, as well as being equipped for the monolithic integration of a large number of transistors that provide array implementation for parallel analyte detections, is a promising candidate to develop PoC devices. It is notable that although incorporating CMOS technology in biosensor design has an outstanding and undeniable impact on system performance, challenges remain in designing biosensors. These challenges essentially include the interface design that couples the assay to the integrated chip, which generally requires additional post-fabrication processes to boost compatibility in sensing modalities [7,8,9]. CMOS technology enables the bulky and expensive instrumentation to be effectively integrated into a low-cost chip, and providing an accurate platform to measure physical changes comes from biological activity. CMOS sensors can be classified into various types based on the detection systems such as electro-chemical [10,11,12,13,14], optical [15,16,17,18,19], magnetic [20,21,22,23,24,25,26,27,28,29,30,31], mechanical [32,33], and thermal [34,35,36,37].

One of the wildly used blocks of the CMOS sensor structure is the inductor–capacitor (LC) oscillator, which can be employed in the sensing, receiving, or transmitting part. For instance, the main core of the frequency-shift-based magnetic sensors is realized with the LC oscillator [20,24,28,29,31], and this oscillator works as a sensing element. In this type of sensor, during the detection process of magnetic nanoparticles, the effective inductance of the resonator changes, which subsequently leads to a resonant frequency down-shift of the LC oscillator. It is noteworthy to mention that due to the fact that there is no consideration for robust operation due to the process, voltage, and temperature (PVT) variations, the oscillation frequency is sensitive to environmental perturbations, which can lead to undesired frequency drifts and impacts of the detection sensitivity on the oscillation frequency shift. In order to achieve robust performance, the oscillator should be designed for the worst-case condition that consequently results in high power consumption.

Another example of sensors for the investigation of the critical role of LC oscillators is nuclear magnetic resonance (NMR) sensors. The main building block of an NMR system is a magnet to produce a static magnetic field, a radio-frequency (RF) coil surrounding a sample, and an RF transceiver to generate excitation RF magnetic fields and monitor the resonance [38]. In NMR sensors, the LC oscillator can be utilized in the RF transceiver [21,23]. For instance, in the NMR system that is reported by Dreyer et al. [31], a VCO is utilized in the phase-locked loop (PLL) as a frequency synthesizer for RF implementation of the transceiver. However, the PLL guarantees the robust performance of the VCO, and it consumes lots of power and area.

VCO-based capacitive sensors are often utilized for measuring complex permittivity and conducting dielectric spectroscopy [39,40,41]. In most structures of VCO-based capacitive sensors, the VCO plays the role of sensing by exposing the tank capacitance to the samples. In the design of such sensors, it should be considered that a single VCO is highly sensitive to variations in PVT, so the sensing results can be affected by environmental effects. In order to overcome this issue, a differential technique such as the utilization of two VCOs as a sensing and reference and differentiating their output frequencies can be employed to enhance the performance of the sensors. It is important to note that the two VCOs should be similar for achieving proper operation. So, this technique, besides consuming more power compared to a single VCO, suffers from a mismatch between two VCOs. In the work in [39], presented by Helmy et al., a VCO is employed to detect the dielectric constant of organic chemicals. When the tank capacitance of the VCO is exposed to the liquid, the oscillation frequency shift will be proportional to the dielectric constant of organic chemicals. In this design [39], the VCO is embedded inside a power-hungry block of a PLL to achieve self-sustained operation.

It is noteworthy that the application of VCOs extends well beyond CMOS sensors, playing a critical role in various systems [42,43,44,45,46,47]. For instance, Lee et al. [46] reported an inductively powered scalable 32-channel wireless neural recording system-on-chip (SoC) for neuroscience applications. In this work, the open-loop VCO is employed to upconvert the baseband pulse width modulation (PWM)–time division multiplexer (TDM) signal through frequency-shift keying (FSK) and is then amplified through RF and transmitted through a miniature wideband monopole antenna. As another example, in [42], Abdelhalim et al. proposed a 915 MHz frequency-shift keying/on–off keying (FSK/OOK) wireless neural recording SoC that utilized a VCO embedded in a PLL to generate stable and tunable RF signals essential for wireless communication. In this design, the closed-loop PLL transmitter prevents the VCO frequency from drifting by ensuring the output frequency is locked to a precise reference crystal oscillator, but it suffers from high power consumption.

During recent decades, a low-voltage and low-power design of VCOs has continuously drawn intensive research. To achieve low power consumption, shrinking the device size for low-voltage operation has become a popular solution. Continuous scaling in device dimensions makes sub-micrometer CMOS transistors susceptible to reliability issues such as channel hot-electron degradation, gate dielectric breakdown, and bias temperature instability [48,49,50,51,52]. These effects cause performance degradation in circuits over time, raise the transistor threshold voltage ($V_{th}$), and drop the electron mobility (${µ}_{n}$), which ultimately results in circuit malfunction [53,54,55]. Some approaches have been developed to mitigate the performance challenges of device reliability. Overdesigning is one of the approaches that is used in the design of circuits for consistent reliability performance. However, this approach increases the power dissipation and the chip area. So, it cannot be accepted as a beneficial solution. Recently, many papers have been reported in the literature to reduce the circuit overdesign while increasing its robustness against reliability effects [56,57,58,59,60,61]. For instance, in [59], Yuan et al. proposed an adaptive gate–source biasing circuit to keep the overdrive voltage of a metal–oxide–semiconductor field-effect transistor (MOSFET) constant, and its performance was then verified for the reliability design of a class AB power amplifier. The presented result shows that the use of an adaptive gate–source biasing circuit makes the power-added efficiency of the power amplifier robust against the threshold voltage and mobility variations.

Another approach utilizes a body bias scheme to improve process variability and circuit reliability [60,61]. The reliability analysis of an MOS varactor for a CMOS LC cross-couple VCO is investigated in [62] by Liu. Based on our knowledge, the impact of the variability and reliability of cross-coupled transistors that provide negative impedance is not examined. In the VCO structure, transconductance of the cross-coupled pair transistors reduces when subjected to a threshold voltage shift and electron mobility degradation, which lead to increases in the phase noise and may cause the oscillation to stop [63,64]. The design of the VCO for low-voltage and low-power applications becomes more challenging and the circuit response will be more sensitive to reliability issues. In RF circuits, there has been active research on low-power low-voltage VCOs [65,66,67,68] and each method has its own advantages and disadvantages.

In this work, an adaptive body basing (ABB) circuit is presented for the design of a 0.6 V LC VCO with a reliability that effectively suppresses the threshold voltage shift and the electron mobility degradation. This paper is organized into four subsequent sections. In Section 2, the proposed structure is presented. In the next section, post-layout simulation results using 180 nm CMOS technology are presented. Finally, the post-fabrication practical consideration and conclusions are presented in Section 4 and Section 5, respectively.

## 2. Circuit Design

### 2.1. Proposed Low-Voltage VCO Design

**Figure 1:**
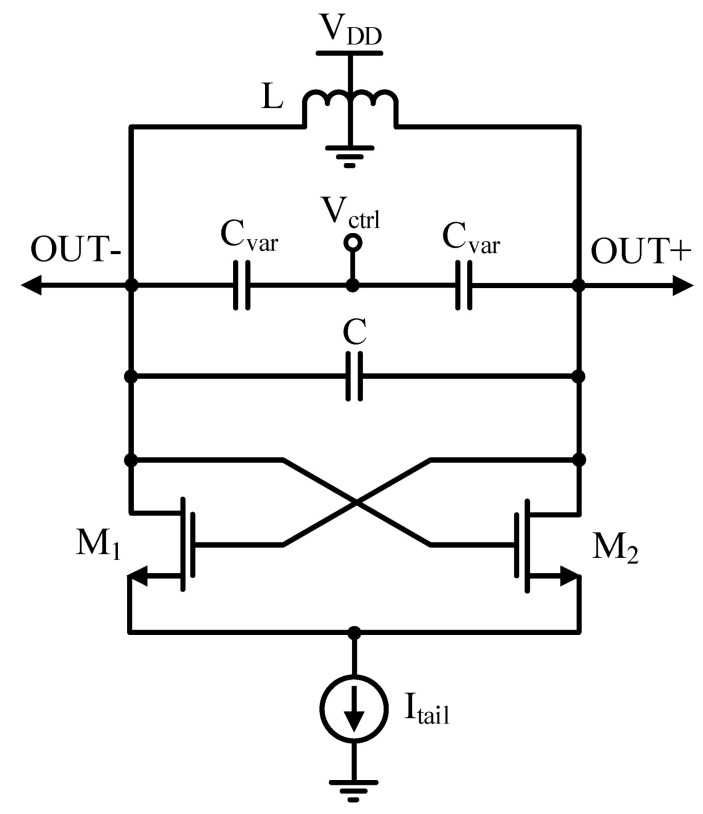
Conventional cross-coupled LC-VCO with a tail current source.

A widely known oscillator structure is the cross-coupled VCO, which is shown in Figure 1. The cross-coupled transistors M_1_ and M_2_ provide an equivalent negative resistance to neutralize the resistive loss in the LC tank circuit.

The design of the VCO based on the tail current source prevents circuit performance variations due to bias, device, and process variations, but results in a higher power dissipation and degraded phase noise due to the flicker noise up-conversion. On the other hand, due to the limited voltage headroom by the tail current source, this VCO will not exhibit the appropriate performance in low-supply-voltage operation.

**Figure 2:**
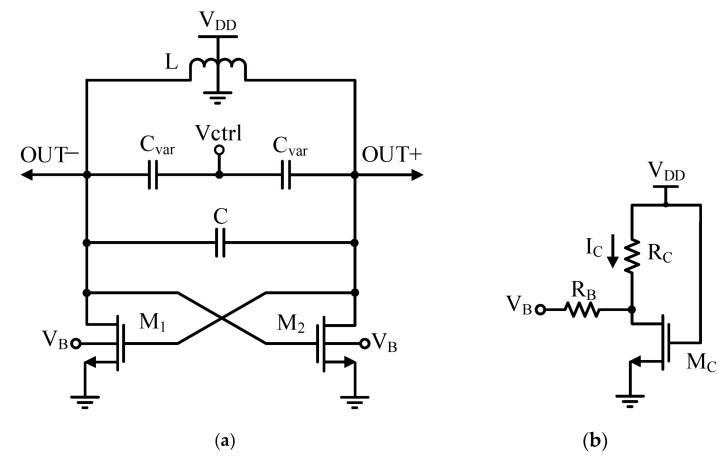
Proposed VCO: (**a**) VCO; (**b**) ABB circuit.

In this paper, an ABB circuit is used, which, in addition to helping to eliminate the tail current source and low-voltage operation, contributes to the reliability performance of the VCO. Figure 2 shows the proposed VCO that consists of the conventional VCO topology with an ABB circuit. The voltage $V_{B}$ feeds back from the ABB circuit to the body of VCO transistors, which acts as a controlling signal and changes their threshold voltage for compensating the $V_{th}$ variation effects of VCO transistors. To further understand the behavior of the proposed VCO, let us explain the operation as follows: When the $V_{th}$ of M_1_(M_2_) increases, the current I_C_ will decrease and lead to the increase in the voltage $V_{B}$. As a result, the $V_{th}\;$of M_1_(M_2_) will decrease and the compensation process arising from reliability degradation will take place. Also, a similar process for the compensation of electron mobility shift takes place, which is illustrated in the following analytical equations.

### 2.2. Analytical Equations

The start-up oscillation condition is given by the following equation [69]:

(1) $${|-2/g}_{m}|\leq \;R_{P}$$

where $g_{m}$ and $R_{P}$ are the transconductance of transistor M_1_(M_2_) and the loss of the LC tank, respectively. $g_{m}$ can be expressed as [70]

(2) $$g_{m}={µ}_{n}\;C_{ox}\;\left(\frac{W}{L}\;\right)\left(V_{GS}-V_{th}\right)=\beta \left(V_{GS}-V_{th}\right)$$

Due to the body effect, the $V_{th}$ of M_1_(M_2_) is well known as [70]

(3) $$V_{th}=V_{th0}+\gamma \left(\sqrt{2\varphi_{f}-V_{bs}}-\sqrt{2\varphi_{f}}\right)=V_{th0}+\gamma \left(\sqrt{2\varphi_{f}-V_{B}}-\sqrt{2\varphi_{f}}\right)$$

where $V_{th0}$ is the threshold voltage at $V_{bs}$ = 0, γ is the body effect coefficient, $\varphi_{f}$ is the bulk Fermi potential, and $V_{bs}$ is the voltage between the body and source terminal. According to (3), $V_{th}$ can be varied by changing ${\;V}_{B}$, where an adaptive threshold voltage variation of VCO transistors can be obtained.

Now, inserting (3) in (2) results in the following:

(4) $$g_{m}=\beta \;(V_{GS}-V_{th0}-\gamma (\sqrt{2\varphi_{f}-V_{B}}+\sqrt{2\varphi_{f}})=g_{m0}-\beta \;\gamma (\sqrt{2\varphi_{f}-V_{B}}+\sqrt{2\varphi_{f}})$$

Examining Figure 2b, the equation for $V_{B}$ is given as

(5) $${R_{C}I}_{C}+V_{B}=V_{DD}$$

The DC current across the M_C_ transistor is expressed as [70]

(6) $$I_{C}\approx \frac{1}{2}\;\mu_{n}Cox\;{\left(\frac{W}{L}\right)}_{C}\;{\left(V_{GS}-V_{thC}\right)}^{2}\;\approx \frac{\beta_{C}}{2}{\left(V_{DD}-V_{thC}\right)}^{2}$$

where $\beta_{C}$ and $V_{thC}$ are the MOSFET structure coefficient and threshold voltage of M_C_, respectively. Replacing $I_{C}$ from (6) into (5) gives

(7) $$V_{B}=V_{DD}-\frac{\beta_{C}}{2}{\;R}_{C}\;{\left(V_{DD}-V_{thC}\right)}^{2}$$

In (7), the deviation in $V_{B}$ because of the threshold voltage variation maintains the following relationship:

(8) dVB=dVBdVthCdVthC=βCRCVDD−VthC dVthC

So, the variations in $g_{m}$ as a result of the body effect can be obtained as (9).

(9) $$dg_{m}=dg_{m0}+\gamma \beta \;\frac{dV_{B}}{2\sqrt{2\varphi_{f}-V_{B}}}$$

Now, inserting (8) in (9) results in the following:

(10) $$dg_{m}=dg_{m0}+\;\frac{dV_{thC}}{2\sqrt{2\varphi_{f}-V_{B}}}{\;\times \;\beta \gamma \beta }_{C}R_{C}\left(V_{DD}-V_{thC}\right)$$

This relation verifies the compensation effect as the first term displays the transconductance shift in M_1_(M_2_) due to $V_{th}$ variations, and the compensation effect resulting from the body bias of M_1_(M_2_) by M_C_ is realized in the second term of (10). So, the transconductance variation due to the threshold voltage degradation and process variation is reduced. The rate of $dg_{m}$ is dependent on γ, $\beta_{C}\;$of the M_C_ transistor, β of VCO transistors, and $R_{C}$, which should be carefully determined to achieve a better design.

The VCO performance is also sensitive to the mobility drift. When the mobility reduces, the transconductance of the VCO thus decreases. The $V_{B}$ fluctuation resulting from mobility degradation by using $V_{B\;}$ from (7) can be derived as (11):

(11) dVB=dVBdβCdβC=−12 RC (VDD−VthC)2 dβC

The transconductance fluctuation subject to dβ and $dV_{B}$ is given by (12), each term of which can be expressed as (13) and (14).

(12) dgm=dgmdβdβ+dgmdVBdVB

(13) dgmdβ=VGS−Vth0−γ(2φf−VB+2φf)

(14) dgmdVB=γβ dVB22φf−VB

By using (11)–(14), the transconductance variation can be written as (15):

(15) $$g_{m}={[\;V}_{GS}-V_{th0}-\gamma (\sqrt{2\varphi_{f}-V_{B}}+\sqrt{2\varphi_{f}})]\;d\beta -\beta \;\frac{\gamma {\;R}_{C}}{4\sqrt{2\varphi_{f}-V_{B}}}\;{\left(V_{DD}-V_{thC}\right)}^{2}\;d\beta_{C}$$

The electron mobility reduction of the VCO transistor decreases the transconductance of M_1_(M_2_), while the reduction in M_C_’s electron mobility increases the $V_{B}$ voltage, which increases the transconductance of VCO, thus finally maintaining a stable transconductance of M_1_(M_2_). So, the introduced ABB besides suppressing $V_{th}$ variations can degrade the mobility variation.

Since phase noise is an important issue in VCO design, studying phase noise is important [71,72,73]. The phase noise of an LC oscillator at an offset frequency of Δ*f* from the frequency of oscillation $f_{0}$ normalized with respect to the carrier is given by [73]:

(16) $$L\left(\Delta f\right)=10{log}_{10}[\frac{F.K.T}{Q^{2}}.\frac{R_{P}}{V_{PP}^{2}}.(\;{\frac{f_{0}}{∆f})}^{2}]$$

where *F* is the noise factor modeling the noise contribution of the active core, *K* is Boltzmann’s constant, *T* is the absolute temperature, *Q* is the tank’s quality factor, $R_{P}=2\pi f_{0}LQ$ is an equivalent resistor modeling the loss of the LC tank, and $V_{PP\;}$ is the voltage swing. One of the major contributing factors for phase noise is the $V_{PP}$ or, in other words, the transconductance of a transistor. A larger value of transconductance and, consequently, $V_{PP}$ enhances the quality factor of the resonator, which leads to the improvement in phase noise performance [1]. The ABB technique not only results in increased transconductance and thus improves the phase noise performance, but it also helps to reduce the phase noise variations due to $V_{th}$ and ${µ}_{n}$ variations by decreasing the $V_{PP}$ and transconductance drift. The simulation results that validate this issue are given in the following section.

## 3. Post-Layout Simulation Results

**Figure 3:**
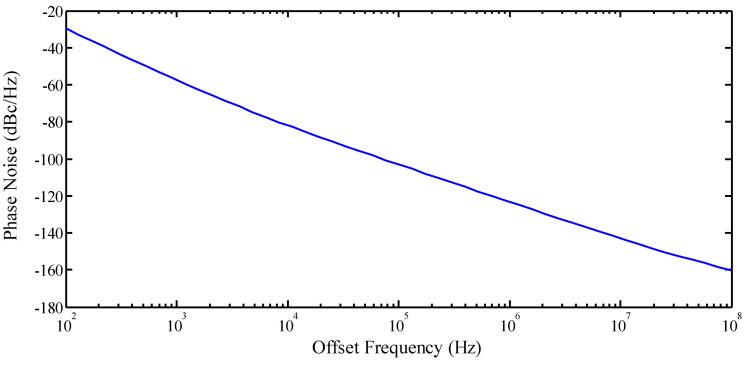
Phase noise of the proposed VCO.

The post-layout simulations of the presented structure are carried out using 180 nm CMOS technology. The dc power consumption of the proposed VCO under 0.6 V of power supply is 398 µW, only 10.9 µW of which is drawn by the ABB circuit. The frequency of the VCO is tunable from 2.379 GHz to 2.461 GHz. Figure 3 shows the phase noise of the VCO for a center frequency of 2.4 GHz. The phase noise at 1 MHz of offset is equal to −123.19 dBc/Hz. It is noteworthy to mention that in order to measure phase noise in practice, a spectrum analyzer [42,74,75] and phase noise meter [76] can be used.

**Figure 4:**
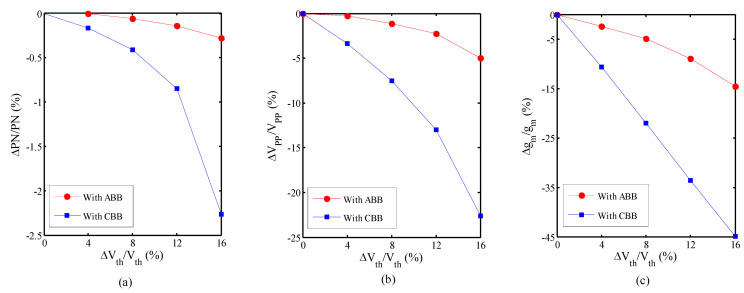
Normalized sensitivity versus normalized threshold voltage variation. (**a**) Phase noise; (**b**) voltage swing; (**c**) transconductance.

**Figure 5:**
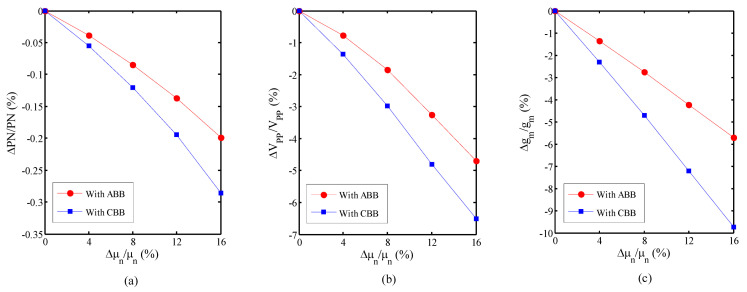
Normalized sensitivity versus normalized electron mobility variation. (**a**) Phase noise; (**b**) voltage swing; (**c**) transconductance.

The VCO is simulated with the proposed ABB circuit and constant body bias (CBB). The threshold voltage and the mobility are independently swept to achieve a normalized phase noise, voltage swing, and transconductance plot versus the normalized threshold voltage, and the mobility drifts and results are displayed in Figure 4 and Figure 5, respectively. Figure 4 shows the normalized sensitivity of the VCO versus the normalized threshold voltage variation. Figure 4a illustrates the normalized phase noise sensitivity of the VCO with the ABB circuit and with the CBB for the 16% normalized threshold voltage shift, which are 0.27% and 2.26%, respectively. The normalized sensitivity of the VCO versus normalized threshold voltage variation for the voltage swing and the transconductance in the 16% normalized threshold voltage shift are 4.97% and 14.6%, respectively, with the ABB circuit and 22.6% and 44.9% with the CBB, respectively. So, the sensitivity of the normalized phase noise, voltage swing, and transconductance of the VCO with the proposed ABB circuit decreases by 8.4 times, 4.55 times, and 3.1 times compared to the CBB, respectively. Also, the normalized sensitivity of the VCO versus the normalized mobility variation with the ABB circuit and with the CBB is shown in Figure 5, which illustrates that the sensitivity of the normalized phase noise, voltage swing, and transconductance of the VCO with the proposed ABB circuit decreases by 1.5 times, 1.4 times, and 1.7 times compared to the CBB, respectively.

**Figure 6:**
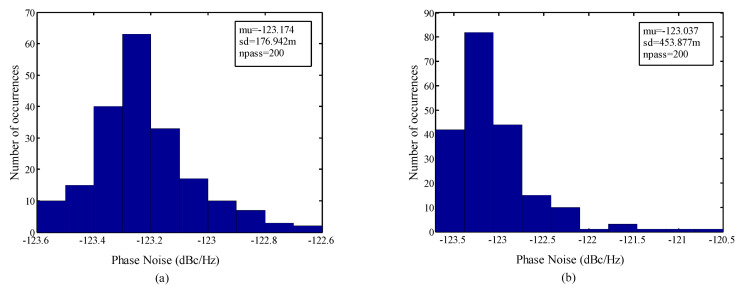
The phase noise Monte Carlo simulation of the VCO with (**a**) proposed ABB and (**b**) CBB.

**Figure 7:**
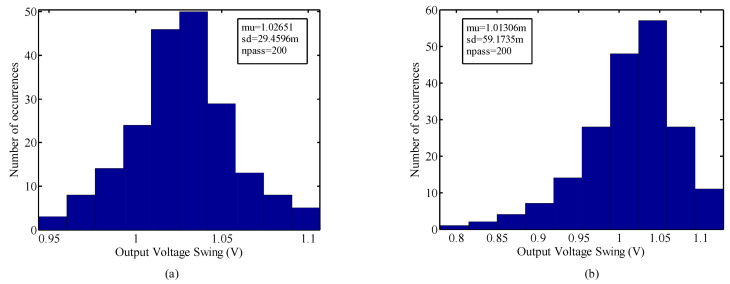
The voltage swing Monte Carlo simulation of the VCO with (**a**) proposed ABB and (**b**) CBB.

**Figure 8:**
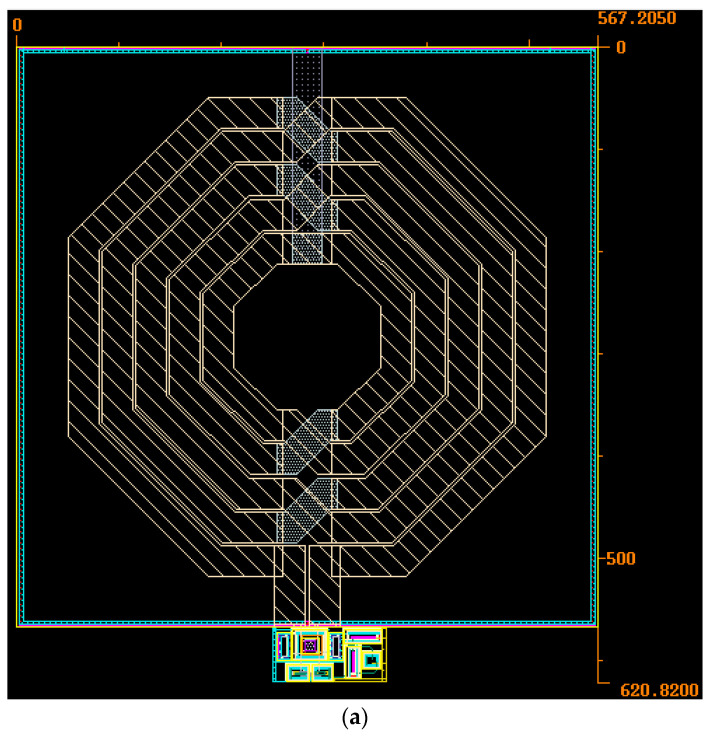

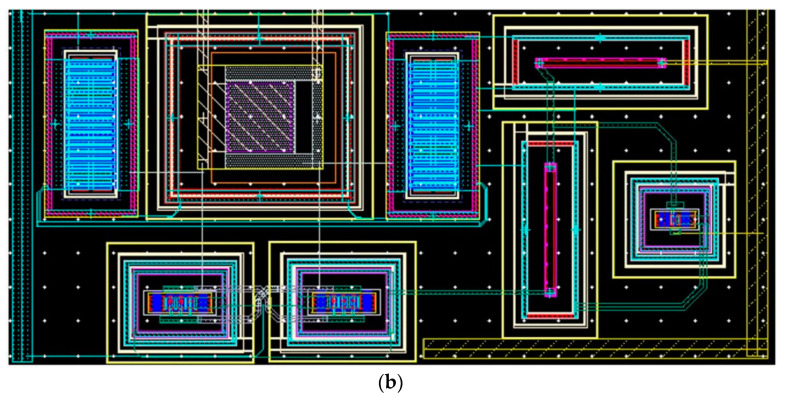
Layout of the VCO with ABB circuit (**a**) with inductor and (**b**) without inductor.

To study the robustness of the structure against process variation, its performance is investigated through Monte Carlo analysis with a run number of 200 and the results are shown in Figure 6 and Figure 7. From Figure 6, the standard deviation of the VCO with the ABB circuit is observed to be less than 177 m, while, in the case of the CBB, it is around 453.87 m. Also, according to Figure 7, the standard deviation of the voltage swing is 29.45 m and 59.17 m with the ABB circuit and CBB, respectively. The promising results from the threshold voltage variation, mobility shift, and Monte Carlo imply the conclusion that the proposed VCO is able to reduce the reliability issue effects and offer an acceptable insensitivity against process variation. The layout and the dimensions of the VCO with the proposed ABB circuit are shown in Figure 8 in which the occupied area is around 0.567 mm × 0.621 mm, and the ABB circuit uses 36.6 µm × 48 µm of this area. The design parameters of the proposed VCO are summarized in Table 1.

To compare the performance of the proposed structure with other VCOs, a figure of merit (FoM) is defined as follows [77]:

(17) $$FoM=L\left(∆f\right)-20\log\left(\frac{f_{0}}{∆f}\right)+10\log\left(\frac{P_{DC}}{1\;mW}\right)$$

where L(Δ*f*) is the phase noise in dBc/Hz at offset frequency Δ*f* from oscillation frequency $f_{0}$ with a DC power consumption of $P_{DC}$ in mW. The results of the circuit simulation in all process corners are summarized in Table 2. It can be seen that the FoM remains relativity constant in the various design corners, which confirms the robustness performance of the designed VCO. The performance of VCO is compared with some other structures in Table 3, which illustrates, in addition to the advantages in terms of power consumption and phase noise, that the proposed VCO also has a better performance compared to the works in the FoM.

## 4. Post-Fabrication Practical Consideration

In this manuscript, we present a comprehensive exploration into the design and analysis of a VCO, elucidating the nuanced advantages inherent in optimal reliability and variability performance through meticulous post-layout simulations. Following the fabrication of the CMOS chip, where the proposed VCO seamlessly integrates with a dedicated CMOS sensor, an imperative facet involves the experimental characterization of reliability and variability performance. This entails a judicious manipulation of the body performance, discerning its impact on phase noise results. This empirical examination serves as a litmus test, unequivocally demonstrating the efficacy of the proposed VCO. Furthermore, the replication of this meticulous evaluation across a myriad of fabricated dies assumes paramount importance. This iterative process serves not only to underscore the resilience of the proposed VCO but also to establish a robust benchmark for reliability and variability performances across diverse chip implementations. The VCO serves as a fundamental building block, seamlessly interfacing with output power amplifier blocks tailored to specific applications. Consequently, the VCO can be strategically linked through a buffer, significantly constraining the requisite power demand. As the primary focus of this paper revolves around the VCO’s design and reliability, a detailed analysis of output power and efficiency will be reserved for subsequent stages of the circuitry. It is noteworthy that the examination of output power and efficiency, as observed in other referenced papers, has been notably sparse, often limited to the characterization of the voltage swing of the output voltage.

## 5. Conclusions

A 0.6 V VCO with a high immunity to threshold voltage variations and electron mobility shift has been proposed. The proposed VCO achieves both low-power consumption and a reliability performance utilizing an ABB circuit. The operational principle of the structure is discussed and its most important relations are derived. The robustness of the structure is examined using the threshold voltage shift, electron mobility decrement, and process variation analysis through Monte Carlo and corner case simulations. Finally, its performance is verified through post-layout simulations in the 180 nm CMOS process. The results show that the sensitivity of the normalized phase noise of the proposed VCO under a 0.16% threshold voltage shift and 0.16% mobility variations decreases by nearly 8.4 times and 1.5 times compared to the CBB, respectively. The VCO has a phase noise of −123.19 dBc/Hz at a 1 MHz offset and provides an FoM of −194.82 dBc/Hz while consuming a low power of 398 µW. The proposed VCO, with its emphasis on low power consumption and exceptional reliability, emerges as a compelling alternative for the design of fully integrated transceivers-on-chip, poised to cater to a diverse range of industrial and medical applications.

## Figures and Tables

**Table 1:** Circuit design parameters.

Parameters	Values	Parameters	Values
**W_M1_/L_M1_**	6 × 2 µm/0.18 µm	**L**	9.46 nH
**W_M1_/L_M1_**	6 × 2 µm/0.18 µm	**R_B_**	20 kΩ
**W_MC_/L_MC_**	1.5 µm/0.18 µm	**R_C_**	25 kΩ
**C**	190 fF		

**Table 2:** VCO performance in different corners.

Corners	^a^ SS	^b^ FF	^c^ FS	^d^ SF
**Phase Noise (dBc/Hz)**	−122.1	−123.18	−123.55	−122.83
**FoM (dBc/Hz)**	−193.52	−193.37	−193.63	−194.65

^a^ Slow NMOS Slow PMOS; ^b^ Fast NMOS Fast PMOS; ^c^ Fast NMOS Slow PMOS; ^d^ Slow NMOS Fast PMOS.

**Table 3:** VCO performance comparisons.

Work	^a^ [65]	^b^ [68]	^b^ [78]	^b^ [79]	^b^ [80]	^b^ This Work
**CMOS Process (nm)**	180	180	180	180	180	180
**Power Supply (V)**	1.1	1.8	1	1.2	0.8	0.6
**Power consumption (mW)**	1.4	1	^c^ 3	1	3.14	0.398
**Oscillation Frequency (GHz)**	2.4	4	5	5	5.4	2.4
**Phase Noise (dBc/Hz) @ 1 MHz**	−122.85	−116.8	−108	−117.7	−119.5	−123.19
**FoM (dBc/Hz)**	−189	−188.9	−177.21	−191.8	−189.18	−194.82

^a^ Measurement; ^b^ Simulation; ^c^ QVCO, half power is taken for evaluation.

## Data Availability

All the relevant data for the simulation and analysis used to conduct this research and generate the results has been included in the paper.

## References

[1] Reddy B., Hassan U., Seymour C., Angus D.C., Isbell T.S., White K., Weir W., Yeh L., Vincent A., Bashir R. (2018). Point-of-care sensors for the management of sepsis. Nat. Biomed. Eng..

[2] John A.S., Price C.P. (2014). Existing and emerging technologies for point-of-care testing. Clin. Biochem. Rev..

[3] Deng W., Wang L., Song S., Zuo X. (2016). Biosensors in POCT application. Prog. Chem..

[4] Rusling J.F., Kumar C.V., Gutkind J.S., Patel V. (2010). Measurement of biomarker proteins for point-of-care early detection and monitoring of cancer. Analyst.

[5] Baryeh K., Takalkar S., Lund M., Liu G. (2017). Medical Biosensors for Point of Care (POC) Applications.

[6] Chen Y.T., Lee Y.C., Lai Y.H., Lim J.C., Huang N.T., Lin C.T., Huang J.J. (2020). Review of integrated optical biosensors for point-of-care applications. Biosensors.

[7] Wang H. Integrated biosensors in CMOS. Proceedings of the 2011 IEEE 54th International Midwest Symposium on Circuits and Systems (MWSCAS).

[8] Singh R., Manickam A., Hassibi A. CMOS biochips for hypothesis-driven DNA analysis. Proceedings of the 2014 IEEE Biomedical Circuits and Systems Conference (BioCAS) Proceedings.

[9] Jang B., Hassibi A. (2009). Biosensor systems in standard CMOS processes: Fact or fiction?. IEEE Trans. Ind. Electron..

[10] Ghafar-Zadeh E., Sawan M., Chodavarapu V.P., Hosseini-Nia T. (2010). Bacteria growth monitoring through a differential CMOS capacitive sensor. IEEE Trans. Biomed. Circuits Syst..

[11] Couniot N., Francis L.A., Flandre D. (2015). A 16×__16 CMOS capacitive biosensor array towards detection of single bacterial cell. IEEE Trans. Biomed. Circuits Syst..

[12] Senevirathna B.P., Lu S., Dandin M.P., Basile J., Smela E., Abshire P.A. (2018). Real-time measurements of cell proliferation using a lab-on-CMOS capacitance sensor array. IEEE Trans. Biomed. Circuits Syst..

[13] Tabrizi H.O., Farhanieh O., Owen Q., Magierowski S., Ghafar-Zadeh E. (2021). Wide input dynamic range fully integrated capacitive sensor for life science applications. IEEE Trans. Biomed. Circuits Syst..

[14] Tabrizi H.O., Forouhi S., Ghafar-Zadeh E. (2022). A High Dynamic Range Dual 8× 16 Capacitive Sensor Array for Life Science Applications. IEEE Trans. Biomed. Circuits Syst..

[15] Manickam A., Singh R., McDermott M.W., Wood N., Bolouki S., Naraghi-Arani P., Johnson K.A., Kuimelis R.G., Schoolnik G., Hassibi A. (2017). A fully integrated CMOS fluorescence biochip for DNA and RNA testing. IEEE J. Solid-State Circuits.

[16] Adamopoulos C., Zarkos P., Buchbinder S., Bhargava P., Niknejad A., Anwar M., Stojanović V. (2021). Lab-on-Chip for Everyone: Introducing an Electronic-Photonic Platform for Multiparametric Biosensing Using Standard CMOS Processes. IEEE Open J. Solid-State Circuits Soc..

[17] Hong L., Li H., Yang H., Sengupta K. (2018). Integrated angle-insensitive nanoplasmonic filters for ultraminiaturized fluorescence microarray in a 65 nm digital CMOS process. ACS Photonics.

[18] Hong L., Li H., Yang H., Sengupta K. (2017). Fully integrated fluorescence biosensors on-chip employing multi-functional nanoplasmonic optical structures in CMOS. IEEE J. Solid-State Circuits.

[19] Eltoukhy H., Salama K., El Gamal A. (2006). A 0.18 μm CMOS bioluminescence detection lab-on-chip. IEEE J. Solid-State Circuits.

[20] Sideris C., Hajimiri A. An integrated magnetic spectrometer for multiplexed biosensing. Proceedings of the 2013 IEEE International Solid-State Circuits Conference Digest of Technical Papers.

[21] Handwerker J., Pérez-Rodas M., Beyerlein M., Vincent F., Beck A., Freytag N., Yu X., Pohmann R., Anders J., Scheffler K. (2020). A CMOS NMR needle for probing brain physiology with high spatial and temporal resolution. Nat. Methods.

[22] Lei K.M., Ha D., Song Y.Q., Westervelt R.M., Martins R., Mak P.I., Ham D. (2020). Portable NMR with parallelism. Anal. Chem..

[23] Dreyer F., Yang Q., Krüger D., Anders J. A chip-based NMR relaxometry system for point-of-care analysis. Proceedings of the 2022 IEEE Biomedical Circuits and Systems Conference (BioCAS).

[24] Wang H., Kosai S., Sideris C., Hajimiri A. An ultrasensitive CMOS magnetic biosensor array with correlated double counting noise suppression. Proceedings of the 2010 IEEE MTT-S International Microwave Symposium.

[25] Pai A., Khachaturian A., Chapman S., Hu A., Wang H., Hajimiri A. (2014). A handheld magnetic sensing platform for antigen and nucleic acid detection. Analyst.

[26] Sideris C., Khial P.P., Ling B., Hajimiri A. A 0.3 ppm dual-resonance transformer-based drift-cancelling reference-free magnetic sensor for biosensing applications. Proceedings of the 2018 IEEE International Solid-State Circuits Conference-(ISSCC).

[27] Sun N., Yoon T.J., Lee H., Andress W., Demas V., Prado P., Weissleder R., Ham D. Palm NMR and one-chip NMR. Proceedings of the 2010 IEEE International Solid-State Circuits Conference-(ISSCC).

[28] Wang H., Mahdavi A., Tirrell D.A., Hajimiri A. (2012). A magnetic cell-based sensor. Lab. Chip.

[29] Wang H., Sideris C., Hajimiri A. A frequency-shift based CMOS magnetic biosensor with spatially uniform sensor transducer gain. Proceedings of the IEEE Custom Integrated Circuits Conference 2010.

[30] Lei K.-M., Mak P.-I., Law M.-K., Martins R.P. (2016). A μNMR CMOS Transceiver Using a Butterfly-Coil Input for Integration with a Digital Microfluidic Device Inside a Portable Magnet. IEEE J. Solid-State Circuits.

[31] Wang H., Chen Y., Hassibi A., Scherer A., Hajimiri A. A frequency-shift CMOS magnetic biosensor array with single-bead sensitivity and no external magnet. Proceedings of the 2009 IEEE International Solid-State Circuits Conference-Digest of Technical Papers.

[32] Chalklen T., Jing Q., Kar-Narayan S. (2020). Biosensors based on mechanical and electrical detection techniques. Sensors.

[33] Arlett J.L., Myers E.B., Roukes M.L. (2011). Comparative advantages of mechanical biosensors. Nat. Nanotechnol..

[34] Someya T., Islam A.K.M.M., Sakurai T., Takamiya M. (2019). An 11-nW CMOS temperature-to-digital converter utilizing sub-threshold current at sub-thermal drain voltage. IEEE J. Solid-State Circuits.

[35] Azcona C., Calvo B., Medrano N., Celma S. (2015). 1.2 V–0.18 μm CMOS Temperature Sensors With Quasi-Digital Output for Portable Systems. IEEE Trans. Instrum. Meas..

[36] Pan S., Angevare J.A., Makinwa K.A.A. 5.4 A Hybrid Thermal-Diffusivity/Resistor-Based Temperature Sensor with a Self-Calibrated Inaccuracy of±0.25 °C (3 Σ) from −55 °C to 125 °C. Proceedings of the 2021 IEEE International Solid-State Circuits Conference (ISSCC).

[37] Pan S., Makinwa K.A.A. (2018). A 0.25 mm 2-Resistor-Based Temperature Sensor With an Inaccuracy of 0.12 °C (3$\sigma $) From −55 °C to 125 °C. IEEE J. Solid-State Circuits.

[38] Rabi I.I., Zacharias J.R., Millman S., Kusch P. (1938). A new method of measuring nuclear magnetic moment. Phys. Rev..

[39] Helmy A.A., Jeon H.J., Lo Y.C., Larsson A.J., Kulkarni R., Kim J., Silva-Martinez J., Entesari K. (2012). A self-sustained CMOS microwave chemical sensor using a frequency synthesizer. IEEE J. Solid-State Circuits.

[40] Nehring J., Bartels M., Weigel R., Kissinger D. A permittivity sensitive PLL based on a silicon-integrated capacitive sensor for microwave biosensing applications. Proceedings of the 2015 IEEE Topical Conference on Biomedical Wireless Technologies, Networks, and Sensing Systems (BioWireleSS).

[41] Elhadidy O., Elkholy M., Helmy A.A., Palermo S., Entesari K. (2013). A CMOS fractional-N PLL-based microwave chemical sensor with 1.5% permittivity accuracy. IEEE Trans. Microw. Theory Tech..

[42] Abdelhalim K., Kokarovtseva L., Velazquez J.L.P., Genov R. (2013). 915-MHz FSK/OOK wireless neural recording SoC with 64 mixed-signal FIR filters. IEEE J. Solid-State Circuits.

[43] Lee M.C., Karimi-Bidhendi A., Malekzadeh-Arasteh O., Wang P.T., Do A.H., Nenadic Z., Heydari P. (2019). A CMOS MedRadio Transceiver With Supply-Modulated Power Saving Technique for an Implantable Brain–Machine Interface System. IEEE J. Solid-State Circuits.

[44] Chuo L.X., Shi Y., Luo Z., Chiotellis N., Foo Z., Kim G., Kim Y., Grbic A., Wentzloff D., Kim H.S. 7.4 A 915MHz asymmetric radio using Q-enhanced amplifier for a fully integrated 3 × 3 × 3 mm 3 wireless sensor node with 20 m non-line-of-sight communication. Proceedings of the 2017 IEEE International Solid-State Circuits Conference (ISSCC).

[45] Harrison R.R., Kier R.J., Chestek C.A., Gilja V., Nuyujukian P., Ryu S., Greger B., Solzbacher F., Shenoy K.V. (2009). Wireless neural recording with single low-power integrated circuit. IEEE Trans. Neural Syst. Rehabil. Eng..

[46] Lee S.B., Lee H.-M., Kiani M., Jow U.-M., Ghovanloo M. (2010). An inductively powered scalable 32-channel wireless neural recording system-on-a-chip for neuroscience applications. IEEE Trans. Biomed. Circuits Syst..

[47] Irazoqui-Pastor P., Mody I., Judy J.W. In-vivo EEG recording using a wireless implantable neural transceiver. Proceedings of the First International IEEE EMBS Conference on Neural Engineering.

[48] Bernstein J.B., Gurfinkel M., Li X., Walters J., Shapira Y., Talmor M. (2006). Electronic circuit reliability modeling. Microelectron. Reliab..

[49] Kumar A., Ning T.H., Fischetti M.V., Gusev E. Hot-carrier charge trapping and reliability in high-k dielectrics. Proceedings of the 2002 Symposium on VLSI Technology. Digest of Technical Papers (Cat. No.01CH37303).

[50] Zafar S., Kumar A., Gusev E., Cartier E. (2005). Threshold voltage instabilities in high-k gate dielectric stacks. IEEE Trans. Device Mater. Reliab..

[51] Tahanout C., Tahi H., Nadji B., Hocini L. (2019). Simple and fast simulation approach to investigate the NBTI effect on suspended gate MOS devices. Int. J. Electron. Lett..

[52] Mark W. (2010). Scaled CMOS Technology Reliability Users Guide.

[53] Afacan E., Yelten M.B., Dündar G. Analog design methodologies for reliability in nanoscale CMOS circuits. Proceedings of the 2017 14th International Conference on Synthesis, Modeling, Analysis and Simulation Methods and Applications to Circuit Design (SMACD).

[54] Kerber A., Nigam T. (2018). Bias temperature instability in scaled CMOS technologies: A circuit perspective. Microelectron. Reliab..

[55] Parihar N., Southwick R.G., Wang M., Stathis J.H., Mahapatra S. (2018). Modeling of NBTI kinetics in replacement metal gate Si and SiGe FinFETs—Part-II: AC stress and recovery. IEEE Trans. Electron. Devices.

[56] Chen M.-J., Ho J.-S., Huang T.-H., Yang C.-H., Jou Y.-N., Wu T. (1996). Back-gate forward bias method for low-voltage CMOS digital circuits. IEEE Trans. Electron. Devices.

[57] Kumar S., Handa M., Bhasin H., Kanaujia B.K., Dwari S., Gautam A.K. (2016). Optimized Threshold Voltage Variation for Tunable Body Biasing CMOS Power Amplifier. Wirel. Pers. Commun..

[58] Yuan J.S., Chen S. (2014). Power amplifier resilient design for process and temperature variations using an on-chip PLL sensing signal. Microelectron. Reliab..

[59] Yuan J.S., Tang H. (2008). CMOS RF design for reliability using adaptive gate–source biasing. IEEE Trans. Electron. Devices.

[60] Liu Y., Yuan J.-S. (2011). CMOS RF low-noise amplifier design for variability and reliability. IEEE Trans. Device Mater. Reliab..

[61] Liu Y., Yuan J.-S. (2011). CMOS RF power amplifier variability and reliability resilient biasing design and analysis. IEEE Trans. Electron. Devices.

[62] Liu Y. (2011). Reliability analysis of MOS varactor in CMOS LC VCO. Microelectron. J..

[63] Hajimiri A., Lee T.H. (1999). Design issues in CMOS differential LC oscillators. IEEE J. Solid-State Circuits.

[64] Lee T.H., Hajimiri A. (2000). Oscillator phase noise: A tutorial. IEEE J. Solid-State Circuits.

[65] Cheng K.-W., Chang S.-K., Huang Y.-C. (2019). Low-Power and Low-Phase-Noise Gm-Enhanced Current-Reuse Differential Colpitts VCO. IEEE Trans. Circuits Syst. II Express Briefs.

[66] Azadmousavi T., Aghdam E.N. (2018). A Low Power Current-Reuse LC-VCO with an Adaptive Body-Biasing Technique. AEU-Int. J. Electron. Commun..

[67] Amer A.G., Ibrahim S.A., Ragai H.F. A 1-mW 12-GHz LC VCO in 65-nm CMOS technology. Proceedings of the 2016 IEEE International Conference on Electronics, Circuits and Systems, ICECS 2016.

[68] Jafari B., Sheikhaei S. (2018). Low phase noise LC VCO with sinusoidal tail current shaping using cascode current source. AEU-Int. J. Electron. Commun..

[69] Razavi B. (2011). RF Microelectronics.

[70] Razavi B. (2001). Design of Analog CMOS Integrated Circuits.

[71] Li M., Seok S., Rolland N., Rolland P.-A. (2011). Design, realization and test of a 2.1 GHz ultra-low phase noise oscillator based on BAW resonator. AEU-Int. J. Electron. Commun..

[72] Sadr H.R., Dousti M. (2012). Purification of inductors and improvement of phase noise in monolithic differential LC-VCOs. AEU-Int. J. Electron. Commun..

[73] Hajimiri A., Lee T.H. (1998). A general theory of phase noise in electrical oscillators. IEEE J. Solid-State Circuits.

[74] Kebe M., Sanduleanu M. (2023). A Low-Phase-Noise 8 GHz Linear-Band Sub-Millimeter-Wave Phase-Locked Loop in 22 nm FD-SOI CMOS. Micromachines.

[75] Chee Y.H., Rabaey J.M., Niknejad A. (2006). Ultra Low Power Transmitters for Wireless Sensor Networks. Ph.D. Thesis.

[76] Lavrič A., Batagelj B., Vidmar M. (2022). Calibration of an RF/Microwave Phase Noise Meter with a Photonic Delay Line. Photonics.

[77] Yun S.-J., Shin S.-B., Choi H.-C., Lee S.-G. A 1mW current-reuse CMOS differential LC-VCO with low phase noise. Proceedings of the Solid-State Circuits Conference.

[78] Naseh S., Dooghabadi M.Z., Deen M.J. A low-voltage low-noise superharmonic quadrature oscillator. Proceedings of the 2008 15th IEEE International Conference on Electronics, Circuits and Systems.

[79] Ying W., Qin P., Jin J., Mo T. A 1mW 5GHz current reuse CMOS VCO with low phase noise and balanced differential outputs. Proceedings of the 2011 International Symposium on Integrated Circuits.

[80] Hsu M.-T., Li W.-J., Hsu S.-C. (2016). Design of low phase noise CMOS VCO using cross coupled topology with capacitor feedback. Microelectron. J..

